# Sex-associated differences in routine inflammatory markers and neuromuscular ultrasound measurements in amyotrophic lateral sclerosis: a retrospective cross-sectional study

**DOI:** 10.1080/07853890.2026.2703317

**Published:** 2026-07-22

**Authors:** Jun Wang, Ying Wang, Tianhua Yang, Xinyi Yan, Jialei Luo, Shenghai Wu, Jiahui Tong, Min Zhao, Gaoyi Yang

**Affiliations:** a Department of Rehabilitation Medicine, Hangzhou First People’s Hospital, Affiliated to Westlake University School of Medicine, Hangzhou, Zhejiang, China; b Department of Ultrasonography, Hangzhou First People’s Hospital, Affiliated to Westlake University School of Medicine, Hangzhou, Zhejiang, China; c Department of Clinical Laboratory, Hangzhou First People’s Hospital, Affiliated to Westlake University School of Medicine, Hangzhou, Zhejiang, China

**Keywords:** Amyotrophic lateral sclerosis, sex-associated differences, routine inflammatory markers, neuromuscular ultrasound, cross-sectional study

## Abstract

**Background:**

Amyotrophic lateral sclerosis (ALS) is a progressive neurodegenerative disorder with substantial clinical heterogeneity. Systemic inflammatory markers and neuromuscular ultrasound measurements have both been studied in ALS, but their sex-associated differences within ALS cohorts remain incompletely characterized.

**Objective:**

To examine sex-associated differences in routine inflammatory markers and neuromuscular ultrasound measurements in patients with ALS, and to determine whether these differences persisted after adjustment for available clinical and anthropometric variables.

**Methods:**

In this retrospective cross-sectional study, 135 patients with ALS were included. Routine inflammatory markers, including neutrophils, monocytes, lymphocytes, platelets, and erythrocyte sedimentation rate (ESR), were analyzed alongside quantitative neuromuscular ultrasound measurements. Between-sex comparisons were performed, and false discovery rate correction was applied to account for multiple testing. Multivariable linear regression analyses were performed with adjustment for age, disease duration, body mass index (BMI), ALSFRS-R total score, FVC% predicted, smoking status, hypertension, and diabetes.

**Results:**

Female patients showed lower ALSFRS-R total scores and higher estimated progression rates in unadjusted comparisons, whereas pulmonary function variables did not differ significantly between sexes. After FDR correction, estimated progression rate, neutrophil count, monocyte count, ESR, and masseter muscle thickness remained significantly different between sexes, while the ALSFRS-R difference was borderline significant. In fully adjusted models, female sex was associated with lower neutrophil count, monocyte count, and median nerve cross-sectional area; biceps brachii thickness showed a less stable association after sensitivity analysis. An exploratory secondary analysis showed lower rectus femoris cross-sectional area during thigh-lift in female patients after full adjustment. In the spline sensitivity analysis, this association remained statistically significant but was interpreted cautiously because it was not present in the earlier adjustment models.

**Conclusions:**

Selected routine inflammatory markers and neuromuscular ultrasound measurements differed between male and female patients within this ALS cohort. These findings support consideration of sex and anthropometric context, including body size, when interpreting inflammatory markers and ultrasound-based structural measurements. In the absence of healthy controls, the observed ultrasound differences cannot be attributed specifically to ALS-related biology. Studies with healthy controls, longitudinal functional outcomes, body-composition assessment, and independent multicenter cohorts are needed to clarify the disease-specific and prognostic relevance of these observations.

## 1. Introduction

Amyotrophic lateral sclerosis (ALS) is a progressive neurodegenerative disorder characterized by degeneration of upper and lower motor neurons, leading to muscle weakness, atrophy, and functional decline [1–3]. Beyond motor neuron degeneration, changes in skeletal muscle, metabolism, and systemic inflammation have been reported in ALS and may provide complementary information on the clinical condition of patients [4–8]. In clinical practice, these markers need to be interpreted together with demographic and anthropometric context rather than as isolated indicators.

Routine clinical assessment in ALS usually includes several domains, but individual laboratory or imaging measurements may be influenced by patient-level factors such as sex, age, and body size. Routine inflammatory markers and neuromuscular ultrasound measurements are both accessible in clinical settings, but their interpretation requires attention to biomarker context, standardized ultrasound acquisition, and biological and anthropometric variation [9–11]. In particular, it remains unclear how these laboratory and ultrasound measurements differ between male and female patients with ALS after considering available clinical and body-size-related variables.

Biological sex has been increasingly recognized as a factor associated with clinical characteristics and survival patterns in ALS [12–14]. Previous studies have suggested that male patients may exhibit stronger inflammatory activation and greater metabolic burden, whereas female patients may present with relatively different inflammatory profiles, preserved muscle function in early stages, and earlier bulbar involvement [12,13,15,16]. These observations may be related to differences in hormonal regulation, immune response, body composition, and muscle-nerve biology [17–19]. However, less is known about whether routine inflammatory markers and neuromuscular ultrasound measurements show sex-associated differences within ALS cohorts, particularly after accounting for body size and available clinical variables.

Neuromuscular ultrasound provides a non-invasive and accessible modality for evaluating muscle thickness, cross-sectional area, echo intensity, and peripheral nerve structure, and has been increasingly applied in the clinical assessment of ALS [20–22]. At the same time, routine inflammatory biomarkers, including neutrophils, monocytes, and erythrocyte sedimentation rate (ESR), have been associated with disease activity, systemic inflammatory burden, and functional decline [23–25]. Because ultrasound-derived muscle and nerve measurements may vary with sex, body size, and reference-value context, and because inflammatory profiles may also differ by sex in ALS, describing sex-associated distributions is important before these measures are interpreted in clinical or research settings [26–28].

Therefore, the present study aimed to investigate sex-associated differences in routine inflammatory markers and quantitative neuromuscular ultrasound parameters in patients with ALS. We further evaluated whether these differences were related to functional status, pulmonary function, and estimated disease progression, using ALSFRS-R, spirometric indices, and ALSFRS-R-based progression rate. This study was designed as an exploratory retrospective cross-sectional analysis intended to improve the interpretation of laboratory and ultrasound measurements in ALS, rather than to establish disease-specific mechanisms or longitudinal prognostic biomarkers.

## 2. Materials and methods

### 2.1. Study design and participants

This retrospective cross-sectional study was conducted in accordance with the Declaration of Helsinki and approved by the Institutional Review Board of Hangzhou First People’s Hospital, Affiliated to Westlake University School of Medicine (Approval No. 2025ZN037-1). The requirement for informed consent was waived by the ethics committee because this study analyzed existing clinical records and involved no direct patient intervention.

Patients with ALS who attended the Motor Neuron Disease Center between December 20, 2024 and March 1, 2025 were screened for eligibility. Inclusion criteria were as follows:

- Diagnosis of ALS according to the revised Gold Coast criteria [29];
- Age 18–80 years;
- Available clinical, laboratory, pulmonary function, ALSFRS-R, and neuromuscular ultrasound records required for data linkage and analysis.

Exclusion criteria:

- Presence of other neuromuscular disorders (e.g. myasthenia gravis or inflammatory neuropathies);
- Severe systemic organ disease or malignancy;
- Acute infection or marked systemic inflammation;
- Records insufficient for data linkage or for identifying the main exposure and outcome variables.

### 2.2. Data collection

Clinical, laboratory, pulmonary function, ALSFRS-R, and neuromuscular ultrasound data were extracted from routine medical records from the same clinical assessment period. For each patient, demographic characteristics, disease-related variables, hematological inflammatory markers, ALSFRS-R score, pulmonary function indices, and ultrasound measurements were obtained from the same individual rather than combined from separate patient subsets. All patients were evaluated at the same center, and no data from external institutions were included. Data extraction and entry were independently checked by two researchers, and discrepancies were resolved by reviewing the original medical records.

### 2.3. Clinical, functional, pulmonary, laboratory, and ultrasound variables

To comprehensively characterize ALS patients from the perspectives of functional status, systemic inflammation, and neuromuscular structural changes, we extracted the following categories of clinical variables:

#### 2.3.1. General characteristics and baseline function

General characteristics included age, sex, disease duration, body mass index, and site of onset, classified as upper limb, lower limb, or bulbar onset. Available baseline functional information included Medical Research Council (MRC) muscle strength grade, scored from 0 to 5, and speech status, classified as clear, slurred, or unable to speak. Functional status was further assessed using the revised Amyotrophic Lateral Sclerosis Functional Rating Scale (ALSFRS-R). Estimated disease progression rate was calculated as: progression rate = (48 − ALSFRS-R total score)/disease duration in months. A higher value indicated faster estimated functional decline. Because this index was derived from baseline ALSFRS-R and reported disease duration, it was interpreted as an estimated cross-sectional progression index rather than a directly observed longitudinal decline rate.

#### 2.3.2. Pulmonary function assessment

In the present study, pulmonary function variables were used to characterize baseline respiratory functional status and to provide clinical adjustment variables in regression models. Pulmonary function was assessed during the same clinical assessment period using a Sunvou-CA2122 pulmonary function testing system (Wuxi Sunvou Medical Electronics Co., Ltd., Wuxi, China) according to routine clinical procedures. The analyzed pulmonary function variables included forced vital capacity percent predicted (FVC% predicted), forced expiratory volume in one second percent predicted (FEV1% predicted), vital capacity percent predicted (VC% predicted), and peak expiratory flow percent predicted (PEF% predicted). These variables were recorded as percentage predicted values to reduce the influence of age, sex, height, and body size on absolute pulmonary function measurements. SNIP and peak cough flow were not included because they were not consistently available in the retrospective clinical records.

#### 2.3.3. Hematological inflammatory markers

Hematological inflammatory markers included neutrophil count (NEU), monocyte count (MONO), lymphocyte count (LYM), platelet count (PLT), and erythrocyte sedimentation rate (ESR). Peripheral venous blood samples were collected as part of routine clinical testing during the same clinical assessment period and analyzed in the Department of Clinical Laboratory. NEU, MONO, LYM, and PLT were obtained from complete blood count testing using a Mindray BC-7500 automated hematology analyzer (Shenzhen Mindray Bio-Medical Electronics Co., Ltd., Shenzhen, China), according to the standard operating procedures of the Department of Clinical Laboratory. ESR was measured using a TEST1 automated erythrocyte sedimentation rate analyzer (Alifax, Italy), according to routine clinical laboratory procedures. No flow cytometry, cytokine assay, or experimental immune profiling was performed in this study. These variables were analyzed as routine clinical indicators of systemic inflammatory status rather than as experimental immune biomarkers.

#### 2.3.4. Muscle and nerve ultrasound parameters

All neuromuscular ultrasound (NMUS) examinations were performed in all enrolled patients at the same center by a physician with more than five years of experience in neuromuscular ultrasound, using a Samsung R10 ultrasound system equipped with a linear-array transducer (LA2-14A; Samsung Medison Co., Ltd., Seoul, South Korea). Measurements were obtained using a standardized protocol during the same clinical assessment period. When bilateral measurements were applicable, both sides were measured twice, and the mean value was used for analysis. Probe pressure was minimized during scanning, and imaging settings were kept as consistent as possible across patients. Muscle thickness was recorded in centimeters, echo intensity was recorded in arbitrary units, and nerve cross-sectional area was recorded in square centimeters.

The following quantitative ultrasound measurements were included in the main analysis: biceps brachii thickness (USBT), resting rectus femoris thickness (USRFRT), rectus femoris thickness during thigh-lift (USTLFRMT), rectus femoris cross-sectional area during thigh-lift (USTLFRMCA), median nerve cross-sectional area (USMNCA), ulnar nerve cross-sectional area (USUNCA), masseter muscle thickness (USMT), and tongue muscle thickness (USTMT). These variables were used to describe muscle and peripheral nerve structural characteristics. Echo intensity measurements of the biceps brachii (USBE), first dorsal interosseous muscle (USFIME), and resting rectus femoris (USRFRE) were also recorded and included in additional exploratory analyses, but they were not considered primary structural outcomes.

### 2.4. Measurement consistency and quality control

All ultrasound data were measured by the same trained evaluator. To verify measurement reliability, we randomly selected 20 patients for repeat measurements and calculated the intraclass correlation coefficient (ICC). The results showed that ICC values for USBT, USRFRT, USTLFRMCA, USMT, and USMNCA were all >0.85, indicating good measurement consistency.

During data acquisition, all ultrasound parameters were obtained under standardized depth, gain, and probe pressure settings, and a fixed patient position was used to reduce inter-operator variability.

### 2.5. Restricted cubic spline-adjusted sensitivity analysis

As a sensitivity analysis, restricted cubic spline (RCS) modeling was used to assess whether the observed sex-associated differences were robust to potential nonlinear effects of continuous covariates. This analysis was performed for outcomes that showed sex-associated differences in the fully adjusted linear models, including NEU, MONO, USBT, USMNCA, and USTLFRMCA. In the RCS-adjusted model, age, disease duration, BMI, ALSFRS-R, and FVC% were modeled using restricted cubic splines with three knots, while sex, smoking status, hypertension, and diabetes were retained as categorical variables. Male patients were used as the reference group. The beta coefficient, 95% confidence interval, and *P*-value for female sex were compared between the primary fully adjusted linear model and the RCS-adjusted model to evaluate the stability of the sex effect. The RCS analysis was used as an exploratory sensitivity analysis and was not intended to assess a nonlinear effect of sex, because sex was a binary exposure.

### 2.6. Statistical analysis

Participants were grouped according to biological sex. Continuous variables were first assessed for normality using the Shapiro-Wilk test. Normally distributed variables were summarized as mean and standard deviation, whereas non-normally distributed variables were summarized as median and interquartile range. Categorical variables were summarized as counts and percentages.

Missing data were assessed before statistical analysis. Some functional, pulmonary, and selected ultrasound-derived variables had missing values. Missing values were addressed using multiple imputation during data preparation. The analyses reported in this study were conducted using the completed analytic dataset generated through that procedure.

Between-sex comparisons were performed for demographic, clinical, functional, pulmonary, inflammatory, and ultrasound variables. Welch t-test, Mann-Whitney U test, Pearson chi-square test, Fisher exact test, or Fisher-Freeman-Halton exact test was used as appropriate according to variable distribution and expected cell counts. To account for multiple unadjusted between-sex comparisons, Benjamini-Hochberg false discovery rate correction was applied across all variables listed in Supplementary Table S1. Both raw *P*-values and FDR-adjusted *P*-values were reported. Effect sizes were calculated to complement *P*-values. Cohen’s d was used for normally distributed continuous variables, rank-biserial correlation for non-normally distributed continuous variables, and Cramer’s V for categorical variables. For continuous variables, effect sizes were calculated as female relative to male.

Estimated disease progression rate was calculated as: progression rate = (48 − ALSFRS-R total score)/disease duration in months. Because this variable was derived from baseline ALSFRS-R total score and reported disease duration, it was interpreted as an estimated cross-sectional progression index rather than a directly observed longitudinal progression rate.

Spearman correlation analyses were performed to describe exploratory cross-sectional relationships among demographic characteristics, functional variables, pulmonary function indices, inflammatory markers, and neuromuscular ultrasound measurements. The covariate set for the multivariable regression models was determined on clinical grounds and was not modified on the basis of the correlation results. These analyses were not used for data-driven selection of outcomes or covariates or for optimization of model fit. The observed strong intercorrelations among spirometric indices were interpreted as consistent with the use of FVC% predicted as a single representative pulmonary covariate in Model 3, rather than simultaneous inclusion of several correlated spirometric measures.

Multivariable linear regression analyses were used to examine the association between sex and selected inflammatory or ultrasound outcomes, with male patients used as the reference group. Model 1 adjusted for age and disease duration. Model 2 further adjusted for BMI. Model 3 further adjusted for ALSFRS-R total score, FVC% predicted, smoking status, hypertension, and diabetes mellitus. Estimated progression rate was not entered into the same regression models with ALSFRS-R total score and disease duration because it was derived from these variables.

Potential multicollinearity among the main exposure and covariates included in the fully adjusted multivariable regression models was evaluated using the generalized variance inflation factor (GVIF). For predictors with more than one degree of freedom, GVIF values were standardized using GVIF^(1/(2·Df)). The GVIF diagnostics were based on the predictor set used in Model 3, including sex, age, disease duration, BMI, ALSFRS-R total score, FVC% predicted, smoking status, hypertension, and diabetes mellitus. Model assumptions were assessed through residual diagnostics. All statistical analyses were conducted using R software, version 4.4.0. All tests were two-sided. A raw P value < 0.05 was considered statistically significant before FDR correction.

## 3. Results

### 3.1. Patient characteristics

A total of 135 patients with ALS were included in this study, comprising 87 males and 48 females. Baseline clinical, functional, pulmonary, inflammatory, and neuromuscular ultrasound characteristics stratified by sex are summarized in Table 1. FDR-adjusted *P*-values and effect sizes for unadjusted between-sex comparisons are provided in Supplementary Table S1.

**Table 1.** Baseline clinical, functional, pulmonary, inflammatory, and neuromuscular ultrasound characteristics stratified by sex.

Characteristic	All (*n* = 135)	Male (*n* = 87)	Female (*n* = 48)	*P* value
Demographic and functional characteristics				
Age (years)	51.26 (9.35)	50.60 (9.30)	52.46 (9.41)	0.272
Disease duration (months)	21.00 [12.00, 26.00]	21.00 [14.00, 26.00]	19.00 [12.00, 25.75]	0.471
Body mass index (kg/m²)	22.84 [20.50, 25.11]	23.34 [20.95, 25.62]	21.92 [20.28, 23.49]	0.024
ALSFRS-R total score	33.00 [25.00, 38.00]	34.00 [26.50, 40.00]	30.50 [24.00, 36.00]	0.010
Estimated progression rate	0.79 [0.44, 1.18]	0.69 [0.37, 1.08]	1.00 [0.64, 1.29]	0.003
Clinical categorical variables				
Upper limb MRC grade (minimum side)				0.392
Grade 0	12 (8.9)	6 (6.9)	6 (12.5)	
Grade 1	24 (17.8)	18 (20.7)	6 (12.5)	
Grade 2	43 (31.9)	29 (33.3)	14 (29.2)	
Grade 3	13 (9.6)	10 (11.5)	3 (6.2)	
Grade 4	29 (21.5)	15 (17.2)	14 (29.2)	
Grade 5	14 (10.4)	9 (10.3)	5 (10.4)	
Lower limb MRC grade				0.534
Grade 0	5 (3.7)	3 (3.4)	2 (4.2)	
Grade 1	8 (5.9)	7 (8.0)	1 (2.1)	
Grade 2	14 (10.4)	7 (8.0)	7 (14.6)	
Grade 3	28 (20.7)	16 (18.4)	12 (25.0)	
Grade 4	50 (37.0)	34 (39.1)	16 (33.3)	
Grade 5	30 (22.2)	20 (23.0)	10 (20.8)	
Speech status				0.444
Unable to speak	6 (4.4)	3 (3.4)	3 (6.2)	
Slurred	76 (56.3)	47 (54.0)	29 (60.4)	
Clear	53 (39.3)	37 (42.5)	16 (33.3)	
Site of onset				0.966
Upper limb	32 (23.7)	20 (23.0)	12 (25.0)	
Lower limb	66 (48.9)	43 (49.4)	23 (47.9)	
Bulbar	37 (27.4)	24 (27.6)	13 (27.1)	
Smoking				0.004
No	123 (91.1)	75 (86.2)	48 (100.0)	
Yes	12 (8.9)	12 (13.8)	0 (0.0)	
Drinking				0.089
No	129 (95.6)	81 (93.1)	48 (100.0)	
Yes	6 (4.4)	6 (6.9)	0 (0.0)	
Hypertension				0.384
No	106 (78.5)	66 (75.9)	40 (83.3)	
Yes	29 (21.5)	21 (24.1)	8 (16.7)	
Diabetes mellitus				0.491
No	126 (93.3)	80 (92.0)	46 (95.8)	
Yes	9 (6.7)	7 (8.0)	2 (4.2)	
Pulmonary function				
FVC, % predicted	69.00 [61.36, 86.00]	74.00 [62.76, 85.50]	67.00 [59.73, 87.25]	0.294
FEV1, % predicted	72.00 [65.00, 87.00]	75.00 [65.47, 87.00]	68.59 [62.75, 85.25]	0.195
PEF, % predicted	65.51 (25.36)	68.71 (24.08)	59.70 (26.82)	0.056
VC, % predicted	61.00 [50.89, 80.00]	62.00 [51.75, 80.00]	58.88 [45.40, 79.25]	0.556
Inflammatory markers				
Platelet count (10^9^/L)	214.35 (53.05)	208.30 (51.62)	225.31 (54.37)	0.080
Neutrophil count (10^9^/L)	3.70 [2.90, 4.50]	4.00 [3.30, 4.65]	3.10 [2.48, 3.92]	<0.001
Monocyte count (10^9^/L)	0.40 [0.30, 0.40]	0.40 [0.30, 0.50]	0.30 [0.30, 0.40]	<0.001
Lymphocyte count (10^9^/L)	1.80 [1.40, 2.10]	1.80 [1.50, 2.20]	1.70 [1.40, 2.02]	0.228
Erythrocyte sedimentation rate (mm/h)	2.00 [2.00, 7.00]	2.00 [2.00, 5.00]	4.00 [2.00, 8.25]	0.006
Ultrasound measurements				
Tongue muscle thickness (USTMT), cm	3.10 (0.61)	3.10 (0.58)	3.09 (0.67)	0.934
Masseter muscle thickness (USMT), cm	1.10 [0.90, 1.30]	1.10 [1.00, 1.30]	1.00 [0.80, 1.12]	<0.001
Biceps brachii thickness (USBT), cm	1.60 [1.30, 2.10]	1.80 [1.30, 2.24]	1.59 [1.20, 1.82]	0.068
Biceps brachii echo intensity (USBE), a.u.	78.00 [62.85, 97.00]	78.00 [65.00, 96.00]	77.50 [60.75, 99.15]	0.941
First dorsal interosseous muscle thickness (USFIMT), cm	0.43 [0.33, 0.61]	0.42 [0.32, 0.61]	0.45 [0.36, 0.61]	0.381
First dorsal interosseous muscle echo intensity (USFIME), a.u.	68.00 [47.00, 88.00]	68.00 [47.00, 84.50]	69.00 [47.50, 88.35]	0.472
Resting rectus femoris thickness (USRFRT), cm	1.60 [1.30, 1.90]	1.60 [1.33, 2.00]	1.60 [1.29, 1.73]	0.352
Resting rectus femoris echo intensity (USRFRE), a.u.	70.00 [54.00, 81.60]	68.00 [52.50, 80.00]	75.00 [54.22, 90.57]	0.302
Rectus femoris thickness during thigh-lift (USTLFRMT), cm	2.32 (0.66)	2.37 (0.73)	2.22 (0.51)	0.161
Rectus femoris cross-sectional area during thigh-lift (USTLFRMCA), cm²	7.23 (2.63)	7.55 (2.73)	6.63 (2.36)	0.043
Median nerve cross-sectional area (USMNCA), cm²	0.08 [0.06, 0.10]	0.08 [0.07, 0.10]	0.07 [0.06, 0.09]	0.025
Ulnar nerve cross-sectional area (USUNCA), cm²	0.03 [0.02, 0.04]	0.03 [0.02, 0.04]	0.03 [0.02, 0.04]	0.709

**Note:** Values are presented as mean (SD) for normally distributed continuous variables and median [IQR] for non-normally distributed continuous variables. Normality was assessed using the Shapiro-Wilk test within sex groups. Categorical variables are presented as n (%). Between-sex comparisons were performed using Welch t-test for normally distributed continuous variables, Mann-Whitney U test for non-normally distributed continuous variables, and chi-square test, Fisher exact test, or Fisher-Freeman-Halton exact test with Monte Carlo simulation for categorical variables, as appropriate. Estimated progression rate was calculated as (48 − ALSFRS-R total score) divided by disease duration in months.

**Abbreviations:** ALSFRS-R, Revised Amyotrophic Lateral Sclerosis Functional Rating Scale; MRC, Medical Research Council; FVC, forced vital capacity; FEV1, forced expiratory volume in 1 s; PEF, peak expiratory flow; VC, vital capacity; CSA, cross-sectional area; a.u., arbitrary units; IQR, interquartile range; SD, standard deviation.

In unadjusted comparisons, female patients had a lower body mass index than male patients (21.92 [20.28, 23.49] vs. 23.34 [20.95, 25.62] kg/m^2^, raw *p* = 0.024), although this difference did not remain significant after FDR correction. Female patients also had lower ALSFRS-R total scores (30.50 [24.00, 36.00] vs. 34.00 [26.50, 40.00], raw *p* = 0.010) and higher estimated progression rates (1.00 [0.64, 1.29] vs. 0.69 [0.37, 1.08], raw *p* = 0.003). After FDR correction, the estimated progression rate remained significant, whereas the ALSFRS-R difference was borderline significant. In contrast, age and disease duration did not differ significantly between sexes. The distributions of upper-limb MRC grade, lower-limb MRC grade, speech status, and site of onset were also comparable between male and female patients.

Pulmonary function indices were generally similar between sexes. FVC% predicted, FEV1% predicted, PEF% predicted, and VC% predicted did not show significant between-sex differences after FDR correction. Smoking was more common in male patients and remained significant after FDR correction. Drinking, hypertension, and diabetes did not differ significantly between sexes.

### 3.2. Sex-associated differences in inflammatory and ultrasound measurements

**Figure 1.**
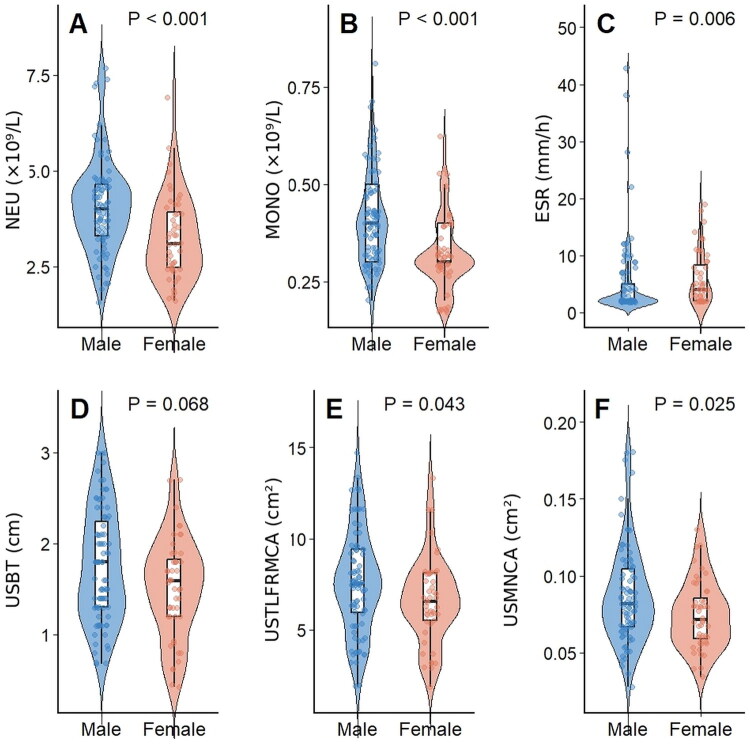
Unadjusted between-sex distributions of representative inflammatory and neuromuscular ultrasound variables. (A) Neutrophil count (NEU, ×10^9^/L); (B) monocyte count (MONO, ×10^9^/L); (C) erythrocyte sedimentation rate (ESR, mm/h); (D) ultrasound-measured biceps brachii thickness (USBT, cm); (E) ultrasound-measured rectus femoris cross-sectional area during thigh-lift (USTLFRMCA, cm^2^); and (F) ultrasound-measured median nerve cross-sectional area (USMNCA, cm^2^). Violin plots show the distribution of each variable by sex, with embedded boxplots indicating the median and interquartile range and points representing individual patients. *P* values shown in the figure are unadjusted between-sex comparison *P* values. FDR-adjusted results are provided in Supplementary Table S1.

Unadjusted group comparisons of selected inflammatory and ultrasound measurements are shown in Figure 1 and summarized in Table 1, with FDR-adjusted results and effect sizes provided in Supplementary Table S1. Female patients had lower NEU levels than male patients (3.10 [2.48, 3.92] vs. 4.00 [3.30, 4.65] × 10^9^/L, raw *p* < 0.001; FDR-adjusted *p* = 0.002). MONO was also lower in female patients (0.30 [0.30, 0.40] vs. 0.40 [0.30, 0.50] × 10^9^/L, raw *p* < 0.001; FDR-adjusted *p* < 0.001). In contrast, ESR was higher in female patients (4.00 [2.00, 8.25] vs. 2.00 [2.00, 5.00] mm/h, raw *p* = 0.006; FDR-adjusted *p* = 0.034). No significant between-sex differences were observed for lymphocyte or platelet counts after FDR correction.

For neuromuscular ultrasound measurements, female patients had lower masseter muscle thickness (USMT) than male patients (1.00 [0.80, 1.12] vs. 1.10 [1.00, 1.30] cm, raw *p* < 0.001; FDR-adjusted *p* = 0.002). Biceps brachii thickness (USBT) tended to be lower in female patients, but the difference did not remain significant after FDR correction. Rectus femoris cross-sectional area during thigh-lift (USTLFRMCA) and median nerve cross-sectional area (USMNCA) were lower in female patients in nominal unadjusted comparisons, but these differences did not remain significant after FDR correction. Other ultrasound parameters, including tongue muscle thickness (USTMT), biceps brachii echo intensity (USBE), first dorsal interosseous muscle thickness (USFIMT), first dorsal interosseous muscle echo intensity (USFIME), resting rectus femoris thickness (USRFRT), resting rectus femoris echo intensity (USRFRE), rectus femoris thickness during thigh-lift (USTLFRMT), and ulnar nerve cross-sectional area (USUNCA), did not show significant between-sex differences after FDR correction.

### 3.3. Correlation analysis of clinical, inflammatory, pulmonary, and ultrasound variables

An exploratory full-variable Spearman correlation analysis was performed to examine the relationships among demographic characteristics, binary clinical variables, ordered functional variables, pulmonary function indices, inflammatory markers, and neuromuscular ultrasound measurements. As shown in Figure 2A, pulmonary function indices were strongly correlated with each other. FVC% predicted was strongly correlated with FEV1% predicted, VC% predicted, and PEF% predicted, with Spearman’s rho values of 0.92, 0.92, and 0.84, respectively. FEV1% predicted was also strongly correlated with VC% predicted and PEF% predicted, with rho values of 0.85 and 0.84, respectively. VC% predicted was positively correlated with PEF% predicted, with rho = 0.79. The significance heatmap in Figure 2B showed that these pulmonary correlations were nominally significant based on unadjusted *P*-values. These correlation patterns were descriptive and were not used to select covariates, optimize model accuracy, or alter the multivariable regression models.

Figure 2.Exploratory Spearman correlation analysis of clinical, inflammatory, pulmonary, and neuromuscular ultrasound variables. (A) Heatmap of Spearman’s correlation coefficients (ρ), showing pairwise correlations among variables. (B) Corresponding heatmap of nominal *P* values for pairwise correlations. In panel B, one asterisk indicates *P* < 0.05, two asterisks indicate *P* < 0.01, and three asterisks indicate *P* < 0.001. Variable abbreviations and labels used in Figure 2 are defined in the Abbreviations section. Correlation analyses were exploratory and intended to describe the cross-sectional correlation structure; they were not used for covariate selection, regression-model optimization, causal inference, or prognostic interpretation.

**Figure:**
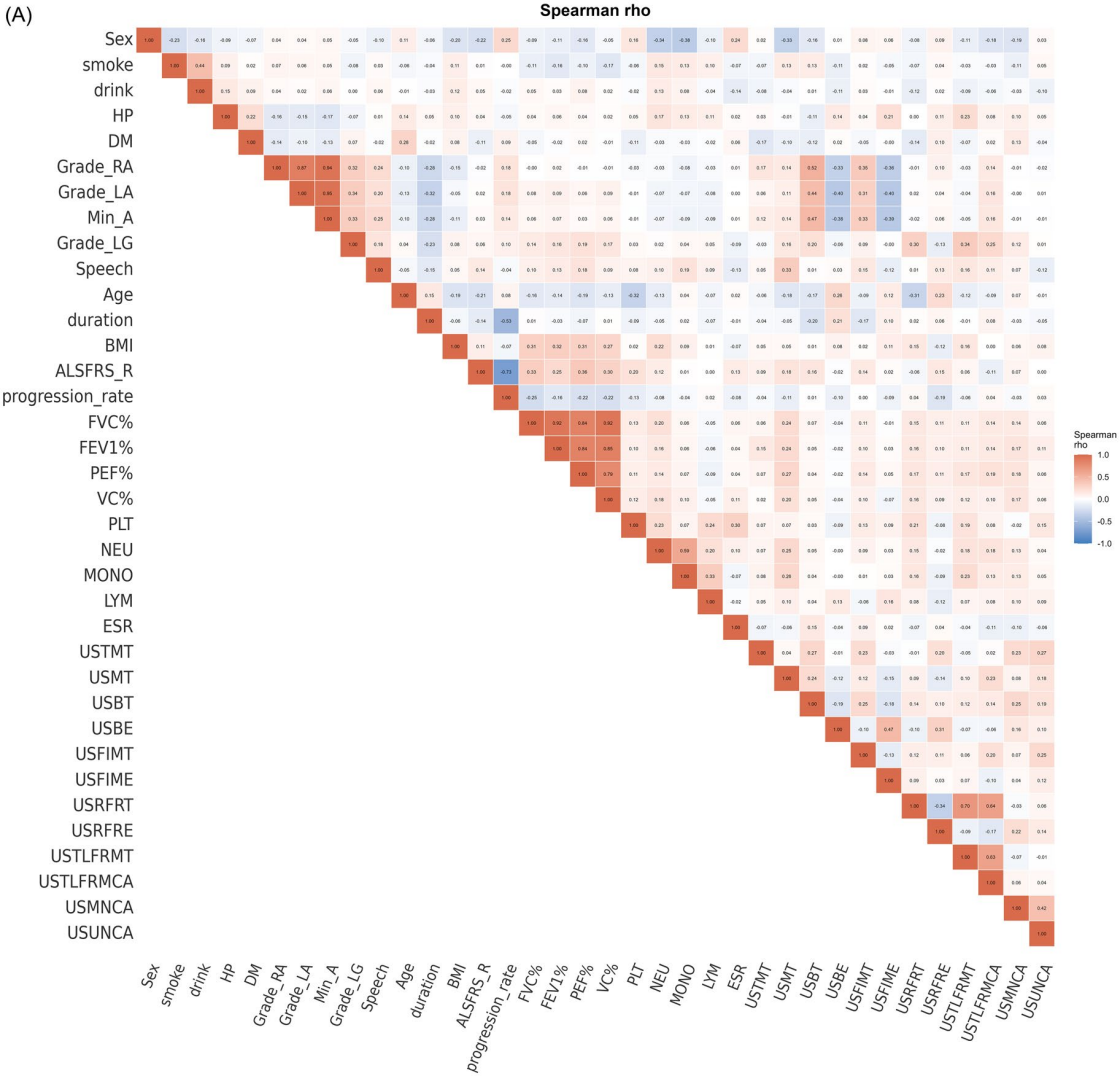

**Figure:**
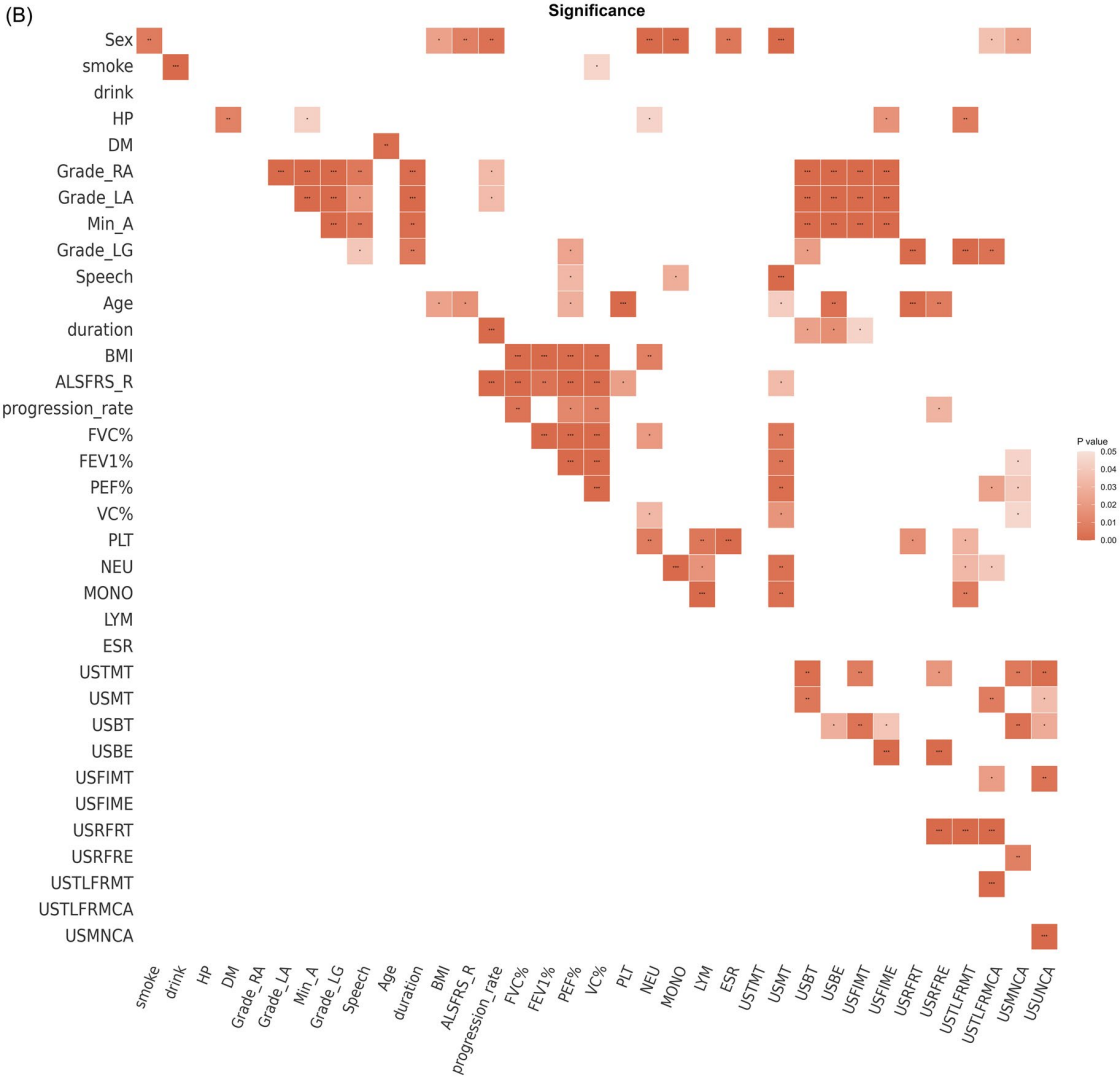

ALSFRS-R total score was strongly and negatively correlated with the estimated progression rate, with rho = −0.73. BMI showed positive correlations with pulmonary function indices, including FVC% predicted, FEV1% predicted, PEF% predicted, and VC% predicted, with rho values of 0.31, 0.32, 0.31, and 0.27, respectively. Correlations involving the sex variable were directionally consistent with the baseline comparisons: female patients tended to have lower BMI, ALSFRS-R total score, NEU, MONO, USMT, USTLFRMCA, and USMNCA, and higher estimated progression rate and ESR.

Among inflammatory markers, NEU and MONO showed a moderate positive correlation, with rho = 0.59. MONO was also positively correlated with LYM, with rho = 0.33, while NEU showed weaker positive correlations with PLT and LYM, with rho values of 0.23 and 0.20, respectively. For neuromuscular ultrasound measurements, several structural parameters showed modest positive correlations. USTMT was positively correlated with USBT and USUNCA, with rho values of 0.27 and 0.27, respectively. USBT was positively correlated with USMNCA and USUNCA, with rho values of 0.25 and 0.19, respectively. USMNCA was moderately correlated with USUNCA, with rho = 0.42. Overall, the correlation structure showed a dense cluster among pulmonary function variables, whereas correlations involving neuromuscular ultrasound measurements were more scattered and generally weaker.

### 3.4. Multivariable regression analysis for inflammatory markers

Multivariable linear regression analyses for inflammatory markers are summarized in Table 2. These analyses were performed to assess whether sex was independently associated with inflammatory markers, with male patients used as the reference group. In Model 1, which adjusted for age and disease duration, female sex was associated with lower NEU levels (β = −0.768, 95% CI −1.188 to −0.349, *p* < 0.001). This association remained significant after further adjustment for BMI in Model 2 (β = −0.686, 95% CI −1.104 to −0.268, *p* = 0.001) and in the fully adjusted Model 3, which additionally included ALSFRS-R total score, FVC% predicted, smoking status, hypertension, and diabetes (β = −0.655, 95% CI −1.096 to −0.214, *p* = 0.004).

**Table 2.** Multivariable linear regression for inflammatory markers.

Outcome	Model	β (95% CI)	*P* value	R^2^
NEU	Model 1	−0.768 [−1.188, −0.349]	<0.001	0.119
NEU	Model 2	−0.686 [−1.104, −0.268]	0.001	0.156
NEU	Model 3	−0.655 [−1.096, −0.214]	0.004	0.194
MONO	Model 1	−0.086 [−0.125, −0.047]	<0.001	0.126
MONO	Model 2	−0.083 [−0.123, −0.044]	<0.001	0.130
MONO	Model 3	−0.092 [−0.133, −0.050]	<0.001	0.168
ESR	Model 1	0.742 [−1.527, 3.011]	0.519	0.004
ESR	Model 2	0.827 [−1.480, 3.134]	0.480	0.005
ESR	Model 3	1.565 [−0.838, 3.969]	0.200	0.074

The dependent variables were NEU, MONO, and ESR. Sex was entered as the main independent variable, with male patients as the reference group. Model 1 adjusted for age and disease duration; Model 2 further adjusted for BMI; Model 3 further adjusted for ALSFRS-R total score, FVC% predicted, smoking status, hypertension, and diabetes mellitus. β values represent the adjusted difference in female patients compared with male patients. ALSFRS-R, Revised Amyotrophic Lateral Sclerosis Functional Rating Scale; BMI, body mass index; ESR, erythrocyte sedimentation rate; FVC, forced vital capacity; MONO, monocyte count; NEU, neutrophil count.

A similar pattern was observed for MONO. Female sex was associated with lower MONO levels in Model 1 (β = −0.086, 95% CI −0.125 to −0.047, *p* < 0.001), Model 2 (β = −0.083, 95% CI −0.123 to −0.044, *p* < 0.001), and the fully adjusted Model 3 (β = −0.092, 95% CI −0.133 to −0.050, *p* < 0.001). In contrast, ESR was not independently associated with sex in any model; in the fully adjusted Model 3, the association between female sex and ESR was not statistically significant (β = 1.565, 95% CI −0.838 to 3.969, *p* = 0.200). These β coefficients represent the adjusted difference in female patients relative to male patients.

### 3.5. Multivariable regression analysis for core neuromuscular ultrasound measurements

Multivariable linear regression analyses for core neuromuscular ultrasound measurements are summarized in Table 3. These analyses were performed to examine the association between sex and core neuromuscular ultrasound measurements, with male patients used as the reference group. Female sex was not independently associated with tongue muscle thickness (USTMT) in any model. In the fully adjusted Model 3, the association between female sex and USTMT remained non-significant (β = −0.013, 95% CI −0.251 to 0.226, *p* = 0.915).

**Table 3.** Multivariable linear regression for core neuromuscular ultrasound measurements.

Outcome	Model	β (95% CI)	*P* value	R^2^
USTMT	Model 1	−0.010 [−0.232, 0.212]	0.932	0.001
USTMT	Model 2	0.001 [−0.225, 0.227]	0.993	0.004
USTMT	Model 3	−0.013 [−0.251, 0.226]	0.915	0.044
USMT	Model 1	−0.139 [−0.268, −0.009]	0.036	0.049
USMT	Model 2	−0.140 [−0.271, −0.008]	0.037	0.049
USMT	Model 3	−0.131 [−0.270, 0.008]	0.064	0.089
USBT	Model 1	−0.235 [−0.442, −0.028]	0.026	0.090
USBT	Model 2	−0.242 [−0.452, −0.031]	0.025	0.092
USBT	Model 3	−0.233 [−0.456, −0.010]	0.041	0.124
USFIMT	Model 1	0.055 [−0.034, 0.144]	0.221	0.061
USFIMT	Model 2	0.051 [−0.040, 0.141]	0.271	0.064
USFIMT	Model 3	0.058 [−0.039, 0.156]	0.238	0.069
USRFRT	Model 1	−0.032 [−0.197, 0.132]	0.698	0.072
USRFRT	Model 2	−0.021 [−0.189, 0.146]	0.800	0.076
USRFRT	Model 3	−0.098 [−0.273, 0.076]	0.267	0.136
USMNCA	Model 1	−0.013 [−0.023, −0.003]	0.010	0.068
USMNCA	Model 2	−0.014 [−0.024, −0.003]	0.010	0.069
USMNCA	Model 3	−0.016 [−0.026, −0.005]	0.005	0.114
USUNCA	Model 1	−0.000 [−0.008, 0.008]	0.931	0.009
USUNCA	Model 2	−0.001 [−0.009, 0.007]	0.833	0.014
USUNCA	Model 3	−0.001 [−0.009, 0.008]	0.855	0.048

The dependent variables were USTMT, USMT, USBT, USFIMT, USRFRT, USMNCA, and USUNCA. Sex was entered as the main independent variable, with male patients as the reference group. Model 1 adjusted for age and disease duration; Model 2 further adjusted for BMI; Model 3 further adjusted for ALSFRS-R total score, FVC% predicted, smoking status, hypertension, and diabetes mellitus. β values represent the adjusted difference in female patients compared with male patients. ALSFRS-R, Revised Amyotrophic Lateral Sclerosis Functional Rating Scale; BMI, body mass index; FVC, forced vital capacity; USBT, biceps brachii thickness; USFIMT, first dorsal interosseous muscle thickness; USMNCA, median nerve cross-sectional area; USMT, masseter muscle thickness; USRFRT, resting rectus femoris thickness; USTMT, tongue muscle thickness; USUNCA, ulnar nerve cross-sectional area.

For masseter muscle thickness (USMT), female sex was associated with lower values in Model 1 (β = −0.139, 95% CI −0.268 to −0.009, *p* = 0.036) and Model 2 (β = −0.140, 95% CI −0.271 to −0.008, *p* = 0.037). However, this association was attenuated after full adjustment in Model 3 and no longer reached statistical significance (β = −0.131, 95% CI −0.270 to 0.008, *p* = 0.064).

For biceps brachii thickness (USBT), female sex was associated with lower values in the three primary linear regression models. The association was observed in Model 1 (β = −0.235, 95% CI −0.442 to −0.028, *p* = 0.026), Model 2 (β = −0.242, 95% CI −0.452 to −0.031, *p* = 0.025), and the fully adjusted Model 3 (β = −0.233, 95% CI −0.456 to −0.010, *p* = 0.041).

Median nerve cross-sectional area (USMNCA) also showed a consistent sex-associated difference. Female sex was associated with lower USMNCA in Model 1 (β = −0.013, 95% CI −0.023 to −0.003, *p* = 0.010), Model 2 (β = −0.014, 95% CI −0.024 to −0.003, *p* = 0.010), and Model 3 (β = −0.016, 95% CI −0.026 to −0.005, *p* = 0.005). No independent association with sex was observed for first dorsal interosseous muscle thickness (USFIMT), resting rectus femoris thickness (USRFRT), or ulnar nerve cross-sectional area (USUNCA) after full adjustment, with Model 3 *P*-values of 0.238, 0.267, and 0.855, respectively.

### 3.6. Additional ultrasound regression analysis

Additional ultrasound measurements were analyzed in Table 4. Echo intensity-related measurements were not independently associated with sex after full adjustment. In Model 3, the associations for biceps brachii echo intensity (USBE), first dorsal interosseous muscle echo intensity (USFIME), and resting rectus femoris echo intensity (USRFRE) were not statistically significant, with *P*-values of 0.765, 0.321, and 0.159, respectively. Rectus femoris thickness during thigh-lift (USTLFRMT) also showed no significant association with sex in Model 3 (β = −0.130, 95% CI −0.378 to 0.117, *p* = 0.300).

**Table 4.** Multivariable linear regression for additional ultrasound measurements.

Outcome	Model	β (95% CI)	*P* value	R^2^
USBE	Model 1	1.537 [−7.811, 10.885]	0.745	0.126
USBE	Model 2	2.656 [−6.782, 12.094]	0.579	0.140
USBE	Model 3	1.510 [−8.467, 11.487]	0.765	0.175
USFIME	Model 1	3.142 [−6.810, 13.095]	0.533	0.058
USFIME	Model 2	4.120 [−5.954, 14.195]	0.420	0.068
USFIME	Model 3	5.275 [−5.195, 15.745]	0.321	0.136
USRFRE	Model 1	3.341 [−5.338, 12.020]	0.448	0.064
USRFRE	Model 2	3.050 [−5.777, 11.876]	0.495	0.065
USRFRE	Model 3	6.565 [−2.607, 15.736]	0.159	0.134
USTLFRMT	Model 1	−0.145 [−0.382, 0.093]	0.231	0.019
USTLFRMT	Model 2	−0.108 [−0.347, 0.130]	0.371	0.044
USTLFRMT	Model 3	−0.130 [−0.378, 0.117]	0.300	0.115
USTLFRMCA	Model 1	−0.842 [−1.778, 0.093]	0.077	0.040
USTLFRMCA	Model 2	−0.872 [−1.824, 0.079]	0.072	0.041
USTLFRMCA	Model 3	−1.202 [−2.196, −0.208]	0.018	0.103

The dependent variables were USBE, USFIME, USRFRE, USTLFRMT, and USTLFRMCA. Sex was entered as the main independent variable, with male patients as the reference group. Model 1 adjusted for age and disease duration; Model 2 further adjusted for BMI; Model 3 further adjusted for ALSFRS-R total score, FVC% predicted, smoking status, hypertension, and diabetes mellitus. β values represent the adjusted difference in female patients compared with male patients. ALSFRS-R, Revised Amyotrophic Lateral Sclerosis Functional Rating Scale; BMI, body mass index; FVC, forced vital capacity; USBE, biceps brachii echo intensity; USFIME, first dorsal interosseous muscle echo intensity; USRFRE, resting rectus femoris echo intensity; USTLFRMCA, rectus femoris cross-sectional area during thigh-lift; USTLFRMT, rectus femoris thickness during thigh-lift.

In the fully adjusted Model 3, rectus femoris cross-sectional area during thigh-lift (USTLFRMCA) was lower in female patients than in male patients (β = −1.202, 95% CI −2.196 to −0.208, *p* = 0.018). However, the associations for USTLFRMCA in Model 1 and Model 2 did not reach statistical significance, with *P*-values of 0.077 and 0.072, respectively. Therefore, USTLFRMCA was interpreted as an exploratory secondary ultrasound finding rather than as a primary or consistently stable result.

### 3.7. RCS-adjusted sensitivity analysis

An RCS-adjusted sensitivity analysis was performed to evaluate whether the observed sex-associated differences remained stable after flexible modeling of continuous covariates. The tested outcomes included NEU, MONO, USBT, USMNCA, and USTLFRMCA. In the RCS-adjusted Model 3, age, disease duration, BMI, ALSFRS-R total score, and FVC% predicted were modeled using restricted cubic splines with three knots, while sex, smoking status, hypertension, and diabetes were retained as categorical variables. Male patients were used as the reference group.

For NEU, the association between female sex and lower values remained significant after RCS adjustment (β = −0.698, 95% CI −1.150 to −0.246, *p* = 0.003). For MONO, the association also remained significant (β = −0.085, 95% CI −0.127 to −0.043, *p* < 0.001). For USMNCA, the association remained stable after RCS adjustment (β = −0.016, 95% CI −0.027 to −0.005, *p* = 0.005). USTLFRMCA also remained lower in female patients in the RCS-adjusted model (β = −1.132, 95% CI −2.159 to −0.105, *p* = 0.031).

For USBT, the direction of the association remained consistent after RCS adjustment, but the association was attenuated and no longer statistically significant (β = −0.201, 95% CI −0.427 to 0.024, *p* = 0.080). Overall, the RCS-adjusted sensitivity analysis supported the direction and statistical significance of the associations for NEU, MONO, and USMNCA. Although USTLFRMCA remained statistically significant in the RCS-adjusted model, it was retained as an exploratory secondary finding because it was not significant in the earlier adjustment models. The association between sex and USBT was more sensitive to flexible adjustment for continuous covariates. Detailed coefficients from the RCS-adjusted sensitivity analysis are provided in Supplementary Table S2. GVIF diagnostics showed low collinearity among the main exposure and covariates included in the fully adjusted models, with all standardized GVIF values below 1.13 (Supplementary Table S3).

## 4. Discussion

### 4.1. Principal findings and scope of interpretation

This retrospective cross-sectional study examined sex-associated differences in routine inflammatory markers and neuromuscular ultrasound (NMUS) measurements in patients with ALS. The main finding is that sex was associated with differences in selected baseline inflammatory and structural measurements, and some of these associations remained after adjustment for disease duration, BMI, functional status, pulmonary function, smoking status, hypertension, and diabetes. In the fully adjusted models, female sex was independently associated with lower NEU and MONO levels, and lower USMNCA showed a relatively consistent model-based ultrasound association. USBT was also lower in female patients in the primary fully adjusted model, although this association was attenuated in the restricted cubic spline sensitivity analysis. USTLFRMCA showed an exploratory secondary association in the additional ultrasound analysis. These findings suggest that sex may need to be considered as a clinical background factor when interpreting routine blood-cell indices and NMUS measurements in ALS, rather than being treated only as a demographic descriptor [12–14,17–19]. In FDR-adjusted unadjusted comparisons, the most consistent between-sex differences were observed for NEU, MONO, ESR, USMT, estimated progression rate, and smoking status. USMNCA and USTLFRMCA did not remain significant after FDR correction in unadjusted comparisons but showed associations in adjusted regression and sensitivity analyses; therefore, they should be interpreted as model-based findings rather than robust unadjusted group differences.

The present findings should be interpreted within the limits of the study design. This study does not establish sex-specific disease mechanisms or provide evidence that these measurements can independently guide prognosis or treatment selection. Instead, the results indicate that routine inflammatory markers and NMUS-derived structural measurements may show sex-associated distributional differences within an ALS cohort. This distinction is important because ALS is clinically heterogeneous, and biological, anatomical, functional, and respiratory factors may influence how clinical measurements should be interpreted [1–3,20–25].

### 4.2. Sex-associated inflammatory patterns

The inflammatory findings may be clinically informative because NEU and MONO are simple, inexpensive, and routinely available blood-cell indices. In this cohort, female patients had lower NEU and MONO levels than male patients, and these associations remained significant after full adjustment. This does not prove that male and female patients have different ALS-specific inflammatory mechanisms. Routine blood-cell counts can be influenced by many factors, including smoking, metabolic status, intercurrent inflammation, medication exposure, and comorbid disease. However, the persistence of these associations after adjustment suggests that sex was associated with differences in the baseline distribution of inflammatory markers in ALS patients. Clinically, this means that higher or lower NEU and MONO values should not be interpreted in isolation. When these markers are used to describe systemic inflammatory status, characterize research cohorts, or support clinical monitoring, sex may need to be considered as part of the interpretive context [15,16,23–25,30,31].

The ESR finding further supports the need for cautious interpretation. ESR was higher in female patients in the unadjusted comparison, but this difference was no longer significant in the fully adjusted model. This pattern suggests that the unadjusted ESR difference may be influenced by other clinical or demographic factors rather than sex alone. Therefore, the inflammatory conclusion should not be stated as a general sex difference across all inflammatory markers. A more accurate interpretation is that NEU and MONO showed stable sex-associated differences, whereas ESR showed only an unadjusted between-sex difference. This distinction is important because overgeneralizing the inflammatory findings could imply a biological mechanism that the present study cannot prove [23–25].

From a rehabilitation-oriented perspective, these routine inflammatory markers may provide contextual information when monitoring fatigue, symptom fluctuation, and recovery after therapeutic activity. However, they should not be used in isolation to determine exercise intensity or rehabilitation strategy. In ALS rehabilitation, inflammatory status should be interpreted together with functional status, respiratory function, nutrition, disease duration, and patient-reported tolerance. This conservative interpretation is more appropriate for a retrospective cross-sectional study and avoids overextending routine laboratory findings into treatment-directing biomarkers [6–8,15,16,25,32].

### 4.3. Neuromuscular ultrasound findings and anatomical confounding

The NMUS results provide a complementary clinical perspective, but they also require careful interpretation. NMUS is non-invasive, repeatable, and increasingly used for assessing muscle and peripheral nerve structure in ALS. In this study, sex-associated differences were not uniform across all ultrasound parameters. In the fully adjusted linear models, USBT and USMNCA were lower in female patients, whereas USTMT, USFIMT, USRFRT, and USUNCA did not show independent sex associations. However, the USBT association did not retain statistical significance after flexible spline adjustment. This selective and partly model-sensitive pattern indicates that sex-related differences should not be assumed for every muscle or nerve site. Each anatomical site should be interpreted according to its measurement characteristics, clinical relevance, and susceptibility to body-size or anatomical confounding [20–22,33].

The association between sex and USBT should be interpreted with particular caution. Biceps brachii thickness is strongly influenced by baseline muscle mass, limb size, and body composition. Although BMI was included in the adjusted models, BMI is not a direct measure of skeletal muscle mass, fat mass, limb length, or regional muscle morphology. The restricted cubic spline sensitivity analysis showed that the direction of the USBT association remained consistent, but the association was no longer statistically significant after flexible modeling of continuous covariates. This suggests that the USBT finding may be more sensitive to modeling assumptions and to the way continuous clinical covariates are handled. Clinically, USBT may still be useful as an accessible structural marker, but sex and body habitus should be considered when interpreting its absolute value. It should not be interpreted as a disease-specific sex effect without longitudinal or control-group evidence [21,25,26,33].

USMNCA showed a relatively consistent model-based association. Female sex was associated with lower USMNCA in the fully adjusted model, and this association remained significant in the restricted cubic spline sensitivity analysis. Median nerve cross-sectional area may reflect a combination of normal anatomical differences, peripheral nerve involvement, chronic denervation-related changes, and technical measurement factors. Because this study did not include healthy controls, we cannot determine whether the lower USMNCA in female patients represents normal sex-related anatomical variation, ALS-related nerve change, or both. Nevertheless, the relative consistency of this association may have interpretive relevance. It suggests that when median nerve CSA is used in ALS assessment or research, sex should be considered during interpretation, especially when comparing patient subgroups or using fixed cutoffs without accounting for demographic and anatomical background [20,22,27,33].

USTLFRMCA was lower in female patients in the additional ultrasound analysis and remained statistically significant in the restricted cubic spline sensitivity analysis. However, this finding should remain exploratory and secondary. The association was not significant in the minimally adjusted models and emerged only after fuller adjustment; the spline result therefore provides supportive rather than confirmatory evidence. USTLFRMCA should be regarded as a hypothesis-generating signal that requires validation rather than as a primary conclusion [21,25,26,33,34].

Across USBT, USMNCA, and USTLFRMCA, the observed sex-associated differences cannot be attributed specifically to ALS-related biology. In the absence of healthy controls, these differences may reflect normal sex-related anatomical variation, disease-related structural changes, or a combination of both.

### 4.4. Functional, respiratory, and correlation context

The addition of ALSFRS-R, estimated progression rate, and pulmonary function variables strengthens the clinical context of this study. Female patients showed lower ALSFRS-R total scores and higher estimated progression rates in unadjusted comparisons, although the ALSFRS-R difference should be interpreted cautiously because it was borderline after FDR correction. Pulmonary function variables were generally comparable between sexes. These results suggest that sex-associated differences in inflammatory and ultrasound variables should be interpreted together with disease severity and respiratory status. In ALS, functional decline and respiratory impairment are central to clinical monitoring. Current guideline-based management emphasizes multidisciplinary care, nutritional support, and timely respiratory support, which is consistent with the inclusion of FVC% predicted as a clinical context variable when interpreting laboratory and ultrasound findings [32,35].

The full-variable correlation analysis provided descriptive context rather than an additional inferential model. Pulmonary function variables formed a strong correlation cluster, indicating substantial overlap among FVC% predicted, FEV1% predicted, VC% predicted, and PEF% predicted. In contrast, correlations involving NMUS measurements were more scattered and generally weaker. NMUS is used to characterize muscle and peripheral nerve structural features in ALS [20–22,33]. These patterns illustrate the degree of overlap among available cross-sectional measures, but they do not establish distinct biological domains, improve prediction, or support treatment selection. The correlation results were not used to select or modify the multivariable regression models.

### 4.5. Rehabilitation-oriented clinical interpretation

The potential clinical relevance of this work lies mainly in contextual interpretation and follow-up monitoring rather than immediate intervention. When clinicians observe lower muscle thickness, smaller nerve cross-sectional area, or differences in inflammatory cell counts, these values should be interpreted alongside sex, body size, disease duration, ALSFRS-R score, pulmonary function, nutritional status, comorbidities, and individual longitudinal change. This is particularly relevant in research cohorts, where sex imbalance may influence cross-sectional group comparisons [20–22,26,27,32,33].

From a rehabilitation perspective, routine inflammatory markers and NMUS-derived structural measurements may provide complementary background information for sex-aware clinical assessment. They should not be used in isolation to determine exercise intensity, rehabilitation load, or treatment strategy; instead, they may complement clinical examination and established functional, respiratory, nutritional, and tolerance assessments. Prospective studies are needed to determine whether sex-specific reference ranges, adjustment strategies, or longitudinal change thresholds improve their clinical interpretation [20–22,32,33,36–38].

### 4.6. Limitations and future directions

Several limitations should be emphasized. First, this was a single-center retrospective cross-sectional study, so it cannot determine whether the observed differences reflect baseline anatomy, different disease trajectories, or different rates of neuromuscular degeneration. Second, the absence of a healthy control group is a major limitation, because normal sex-related anatomical differences cannot be separated from ALS-related structural changes. Third, although the models adjusted for BMI, BMI does not fully account for skeletal muscle mass, fat mass, limb length, or regional body composition. This is especially important for NMUS measurements of muscle thickness and cross-sectional area. Fourth, the sample size was modest for complex adjusted models and restricted cubic spline sensitivity analysis. The spline analysis should therefore be interpreted as supportive and exploratory, not as definitive evidence of nonlinear covariate effects [12–14,21,25–27,33].

An additional limitation concerns missing-data handling. Although missing values were addressed using multiple imputation during data preparation, the analyses reported here were based on the completed analytic dataset rather than on pooled estimates across individual imputed datasets. Accordingly, imputation-related uncertainty may not have been fully reflected in the reported standard errors, confidence intervals, and *P*-values.

Another limitation is that the inflammatory markers were limited to routine laboratory indices. NEU, MONO, LYM, PLT, and ESR are clinically accessible, but they are not specific to ALS-related immune processes. More detailed biomarkers, including cytokines, neurofilament light chain, metabolic markers, and body composition measures, may help clarify the biological meaning of the observed sex-associated differences. NMUS measurements were also obtained from a single center, and external validation is needed to assess whether the same findings can be reproduced using different operators, devices, protocols, and patient populations [6,15,16,39,40].

Replication in independent multicenter cohorts is required before these findings can be generalized beyond the present clinical setting.

Future studies should use longitudinal designs to determine whether these sex-associated differences remain stable over time or predict clinically meaningful outcomes such as ALSFRS-R decline, respiratory deterioration, nutritional decline, mobility loss, or survival. Including healthy controls would help distinguish normal anatomical sex differences from ALS-related structural changes. Future work should also incorporate more direct body composition measures, such as skeletal muscle mass, fat mass, limb circumference, and regional muscle morphology, to reduce residual anatomical confounding. If validated, sex-aware interpretation of inflammatory markers and NMUS measurements may improve the clinical description of ALS heterogeneity and support more precise monitoring strategies in clinical and rehabilitation settings [25,32,33,38–40].

## 5. Conclusion

In conclusion, sex-associated differences were observed in selected routine inflammatory markers and NMUS measurements within this ALS cohort. Female sex was independently associated with lower NEU and MONO levels and lower USMNCA, whereas USBT showed a less stable association after sensitivity analysis. USTLFRMCA showed an exploratory secondary association in the additional analysis. In the absence of healthy controls, the differences in USBT, USMNCA, and USTLFRMCA cannot be attributed specifically to ALS-related biology and may reflect normal sex-related anatomical variation, ALS-related structural changes, or both. These findings should not be interpreted as evidence for sex-specific disease mechanisms or treatment strategies. Rather, they support considering sex as an important clinical context when interpreting routine inflammatory and structural ultrasound measurements in ALS. Prospective studies with healthy controls, longitudinal outcomes, body-composition assessment, and independent multicenter cohorts are needed to validate these observations before broader generalization.

## Supplementary Material

Supplementary_Table_S1.docx

Supplementary_Table_S3.docx

Supplementary_Table_S2.docx

## Data Availability

The datasets generated and/or analyzed during the current study are available from the corresponding author on reasonable request.
