# Supplementary material for: Sex-associated differences in routine inflammatory markers and neuromuscular ultrasound measurements in amyotrophic lateral sclerosis: a retrospective cross-sectional study
Source: Ann Med. 2026 Jul 22;58(1):2703317. doi: 10.1080/07853890.2026.2703317 (PMC13393054; doi:10.1080/07853890.2026.2703317)
Supplement: Supplementary_Table_S1.docx [file IANN_A_2703317_SM1941.docx]

**Supplementary Table S1. FDR-adjusted P values and effect sizes for unadjusted between-sex comparisons**

| **Variable** | **Overall (n=135)** | **Male (n=87)** | **Female (n=48)** | **Test** | **Raw P** | **FDR-adjusted P** | **Significant after FDR** | **Effect size** | **Effect size type** |
| --- | --- | --- | --- | --- | --- | --- | --- | --- | --- |
| Age | 51.26 ± 9.35 | 50.60 ± 9.30 | 52.46 ± 9.41 | Welch t-test | 0.272 | 0.516 | No | 0.199 | Cohen's d |
| Disease duration | 21.00 [12.00, 26.00] | 21.00 [14.00, 26.00] | 19.00 [12.00, 25.75] | Mann-Whitney U | 0.471 | 0.607 | No | -0.075 | Rank-biserial correlation |
| BMI | 22.84 [20.50, 25.11] | 23.34 [20.95, 25.62] | 21.92 [20.28, 23.49] | Mann-Whitney U | 0.024 | 0.099 | No | -0.235 | Rank-biserial correlation |
| ALSFRS-R | 33.00 [25.00, 38.00] | 34.00 [26.50, 40.00] | 30.50 [24.00, 36.00] | Mann-Whitney U | 0.010 | 0.049 | Yes | -0.27 | Rank-biserial correlation |
| Estimated progression rate | 0.79 [0.44, 1.18] | 0.69 [0.37, 1.08] | 1.00 [0.64, 1.29] | Mann-Whitney U | 0.003 | 0.029 | Yes | 0.307 | Rank-biserial correlation |
| PLT | 214.35 ± 53.05 | 208.30 ± 51.62 | 225.31 ± 54.37 | Welch t-test | 0.080 | 0.221 | No | 0.323 | Cohen's d |
| NEU | 3.70 [2.90, 4.50] | 4.00 [3.30, 4.65] | 3.10 [2.48, 3.92] | Mann-Whitney U | <0.001 | 0.002 | Yes | -0.406 | Rank-biserial correlation |
| MONO | 0.40 [0.30, 0.40] | 0.40 [0.30, 0.50] | 0.30 [0.30, 0.40] | Mann-Whitney U | <0.001 | <0.001 | Yes | -0.431 | Rank-biserial correlation |
| LYM | 1.80 [1.40, 2.10] | 1.80 [1.50, 2.20] | 1.70 [1.40, 2.02] | Mann-Whitney U | 0.228 | 0.455 | No | -0.126 | Rank-biserial correlation |
| ESR | 2.00 [2.00, 7.00] | 2.00 [2.00, 5.00] | 4.00 [2.00, 8.25] | Mann-Whitney U | 0.006 | 0.034 | Yes | 0.269 | Rank-biserial correlation |
| FVC% | 69.00 [61.36, 86.00] | 74.00 [62.76, 85.50] | 67.00 [59.73, 87.25] | Mann-Whitney U | 0.294 | 0.518 | No | -0.110 | Rank-biserial correlation |
| FEV1% | 72.00 [65.00, 87.00] | 75.00 [65.47, 87.00] | 68.59 [62.75, 85.25] | Mann-Whitney U | 0.195 | 0.413 | No | -0.135 | Rank-biserial correlation |
| PEF% | 65.51 ± 25.36 | 68.71 ± 24.08 | 59.70 ± 26.82 | Welch t-test | 0.056 | 0.183 | No | -0.360 | Cohen's d |
| VC% | 61.00 [50.89, 80.00] | 62.00 [51.75, 80.00] | 58.88 [45.40, 79.25] | Mann-Whitney U | 0.556 | 0.646 | No | -0.062 | Rank-biserial correlation |
| USTMT | 3.10 ± 0.61 | 3.10 ± 0.58 | 3.09 ± 0.67 | Welch t-test | 0.934 | 0.966 | No | -0.015 | Cohen's d |
| USMT | 1.10 [0.90, 1.30] | 1.10 [1.00, 1.30] | 1.00 [0.80, 1.12] | Mann-Whitney U | <0.001 | 0.002 | Yes | -0.391 | Rank-biserial correlation |
| USBT | 1.60 [1.30, 2.10] | 1.80 [1.30, 2.24] | 1.59 [1.20, 1.82] | Mann-Whitney U | 0.068 | 0.204 | No | -0.190 | Rank-biserial correlation |
| USBE | 78.00 [62.85, 97.00] | 78.00 [65.00, 96.00] | 77.50 [60.75, 99.15] | Mann-Whitney U | 0.941 | 0.966 | No | 0.008 | Rank-biserial correlation |
| USFIMT | 0.43 [0.33, 0.61] | 0.42 [0.32, 0.61] | 0.45 [0.36, 0.61] | Mann-Whitney U | 0.381 | 0.564 | No | 0.091 | Rank-biserial correlation |
| USFIME | 68.00 [47.00, 88.00] | 68.00 [47.00, 84.50] | 69.00 [47.50, 88.35] | Mann-Whitney U | 0.472 | 0.607 | No | 0.075 | Rank-biserial correlation |
| USRFRT | 1.60 [1.30, 1.90] | 1.60 [1.33, 2.00] | 1.60 [1.29, 1.73] | Mann-Whitney U | 0.352 | 0.564 | No | -0.097 | Rank-biserial correlation |
| USRFRE | 70.00 [54.00, 81.60] | 68.00 [52.50, 80.00] | 75.00 [54.22, 90.57] | Mann-Whitney U | 0.302 | 0.518 | No | 0.108 | Rank-biserial correlation |
| USTLFRMT | 2.32 ± 0.66 | 2.37 ± 0.73 | 2.22 ± 0.51 | Welch t-test | 0.161 | 0.386 | No | -0.229 | Cohen's d |
| USTLFRMCA | 7.23 ± 2.63 | 7.55 ± 2.73 | 6.63 ± 2.36 | Welch t-test | 0.043 | 0.156 | No | -0.352 | Cohen's d |
| USMNCA | 0.08 [0.06, 0.10] | 0.08 [0.07, 0.10] | 0.07 [0.06, 0.09] | Mann-Whitney U | 0.025 | 0.099 | No | -0.234 | Rank-biserial correlation |
| USUNCA | 0.03 [0.02, 0.04] | 0.03 [0.02, 0.04] | 0.03 [0.02, 0.04] | Mann-Whitney U | 0.709 | 0.774 | No | 0.039 | Rank-biserial correlation |
| Right upper-limb MRC grade |  |  |  | Fisher-Freeman-Halton exact test | 0.194 | 0.413 | No | 0.233 | Cramer's V |
| Grade 0 | 11 (8.1) | 5 (5.7) | 6 (12.5) |  |  |  |  |  |  |
| Grade 1 | 22 (16.3) | 18 (20.7) | 4 (8.3) |  |  |  |  |  |  |
| Grade 2 | 33 (24.4) | 20 (23.0) | 13 (27.1) |  |  |  |  |  |  |
| Grade 3 | 18 (13.3) | 14 (16.1) | 4 (8.3) |  |  |  |  |  |  |
| Grade 4 | 29 (21.5) | 16 (18.4) | 13 (27.1) |  |  |  |  |  |  |
| Grade 5 | 22 (16.3) | 14 (16.1) | 8 (16.7) |  |  |  |  |  |  |
| Left upper-limb MRC grade |  |  |  | Fisher-Freeman-Halton exact test | 0.618 | 0.696 | No | 0.162 | Cramer's V |
| Grade 0 | 10 (7.4) | 5 (5.7) | 5 (10.4) |  |  |  |  |  |  |
| Grade 1 | 20 (14.8) | 15 (17.2) | 5 (10.4) |  |  |  |  |  |  |
| Grade 2 | 39 (28.9) | 25 (28.7) | 14 (29.2) |  |  |  |  |  |  |
| Grade 3 | 20 (14.8) | 15 (17.2) | 5 (10.4) |  |  |  |  |  |  |
| Grade 4 | 26 (19.3) | 15 (17.2) | 11 (22.9) |  |  |  |  |  |  |
| Grade 5 | 20 (14.8) | 12 (13.8) | 8 (16.7) |  |  |  |  |  |  |
| Minimum upper-limb MRC grade |  |  |  | Fisher-Freeman-Halton exact test | 0.392 | 0.564 | No | 0.199 | Cramer's V |
| Grade 0 | 12 (8.9) | 6 (6.9) | 6 (12.5) |  |  |  |  |  |  |
| Grade 1 | 24 (17.8) | 18 (20.7) | 6 (12.5) |  |  |  |  |  |  |
| Grade 2 | 43 (31.9) | 29 (33.3) | 14 (29.2) |  |  |  |  |  |  |
| Grade 3 | 13 (9.6) | 10 (11.5) | 3 (6.2) |  |  |  |  |  |  |
| Grade 4 | 29 (21.5) | 15 (17.2) | 14 (29.2) |  |  |  |  |  |  |
| Grade 5 | 14 (10.4) | 9 (10.3) | 5 (10.4) |  |  |  |  |  |  |
| Lower-limb MRC grade |  |  |  | Fisher-Freeman-Halton exact test | 0.534 | 0.641 | No | 0.176 | Cramer's V |
| Grade 0 | 5 (3.7) | 3 (3.4) | 2 (4.2) |  |  |  |  |  |  |
| Grade 1 | 8 (5.9) | 7 (8.0) | 1 (2.1) |  |  |  |  |  |  |
| Grade 2 | 14 (10.4) | 7 (8.0) | 7 (14.6) |  |  |  |  |  |  |
| Grade 3 | 28 (20.7) | 16 (18.4) | 12 (25.0) |  |  |  |  |  |  |
| Grade 4 | 50 (37.0) | 34 (39.1) | 16 (33.3) |  |  |  |  |  |  |
| Grade 5 | 30 (22.2) | 20 (23.0) | 10 (20.8) |  |  |  |  |  |  |
| Speech status |  |  |  | Fisher-Freeman-Halton exact test | 0.444 | 0.607 | No | 0.103 | Cramer's V |
| Unable to speak | 6 (4.4) | 3 (3.4) | 3 (6.2) |  |  |  |  |  |  |
| Slurred | 76 (56.3) | 47 (54.0) | 29 (60.4) |  |  |  |  |  |  |
| Clear | 53 (39.3) | 37 (42.5) | 16 (33.3) |  |  |  |  |  |  |
| Site of onset |  |  |  | Pearson chi-square test | 0.966 | 0.966 | No | 0.023 | Cramer's V |
| Upper limb | 32 (23.7) | 20 (23.0) | 12 (25.0) |  |  |  |  |  |  |
| Lower limb | 66 (48.9) | 43 (49.4) | 23 (47.9) |  |  |  |  |  |  |
| Bulbar | 37 (27.4) | 24 (27.6) | 13 (27.1) |  |  |  |  |  |  |
| Smoking |  |  |  | Fisher exact test | 0.004 | 0.032 | Yes | 0.232 | Cramer's V |
| No | 123 (91.1) | 75 (86.2) | 48 (100.0) |  |  |  |  |  |  |
| Yes | 12 (8.9) | 12 (13.8) | 0 (0.0) |  |  |  |  |  |  |
| Drinking |  |  |  | Fisher exact test | 0.089 | 0.228 | No | 0.160 | Cramer's V |
| No | 129 (95.6) | 81 (93.1) | 48 (100.0) |  |  |  |  |  |  |
| Yes | 6 (4.4) | 6 (6.9) | 0 (0.0) |  |  |  |  |  |  |
| Hypertension |  |  |  | Fisher exact test | 0.384 | 0.564 | No | 0.087 | Cramer's V |
| No | 106 (78.5) | 66 (75.9) | 40 (83.3) |  |  |  |  |  |  |
| Yes | 29 (21.5) | 21 (24.1) | 8 (16.7) |  |  |  |  |  |  |
| Diabetes mellitus |  |  |  | Fisher exact test | 0.491 | 0.609 | No | 0.074 | Cramer's V |
| No | 126 (93.3) | 80 (92.0) | 46 (95.8) |  |  |  |  |  |  |
| Yes | 9 (6.7) | 7 (8.0) | 2 (4.2) |  |  |  |  |  |  |

**Notes:** Values are based on the completed analytic dataset generated during the multiple-imputation data-preparation procedure. Values are presented as mean (SD) for normally distributed continuous variables and median [IQR] for non-normally distributed continuous variables. Normality was assessed using the Shapiro-Wilk test within sex groups. Categorical variables are presented as n (%). Between-sex comparisons were performed using Welch t-test for normally distributed continuous variables, Mann-Whitney U test for non-normally distributed continuous variables, and Pearson chi-square test, Fisher exact test, or Fisher-Freeman-Halton exact test with Monte Carlo simulation for categorical variables, as appropriate. FDR-adjusted P values were calculated using the Benjamini-Hochberg method across all variables listed in this table. Effect sizes were reported as Cohen’s d for normally distributed continuous variables, rank-biserial correlation for non-normally distributed continuous variables, and Cramer’s V for categorical variables. For continuous variables, effect sizes were calculated as female relative to male; therefore, negative values indicate lower values in female patients. Cramer’s V is non-directional. Estimated progression rate was calculated as (48 − ALSFRS-R total score) divided by disease duration in months. Supplementary Table S1 includes the minimum upper-limb MRC grade shown in Table 1 and additional side-specific upper-limb MRC grades for completeness.

**Abbreviations:** ALSFRS-R, Revised Amyotrophic Lateral Sclerosis Functional Rating Scale; BMI, body mass index; CSA, cross-sectional area; FDR, false discovery rate; FVC, forced vital capacity; FEV1, forced expiratory volume in 1 second; IQR, interquartile range; MRC, Medical Research Council; PEF, peak expiratory flow; SD, standard deviation; VC, vital capacity.
