# Supplementary material for: Sex-associated differences in routine inflammatory markers and neuromuscular ultrasound measurements in amyotrophic lateral sclerosis: a retrospective cross-sectional study
Source: Ann Med. 2026 Jul 22;58(1):2703317. doi: 10.1080/07853890.2026.2703317 (PMC13393054; doi:10.1080/07853890.2026.2703317)
Supplement: Supplementary_Table_S3.docx [file IANN_A_2703317_SM1940.docx]

**Supplementary Table S3. GVIF diagnostics for the main exposure and covariates included in the fully adjusted multivariable regression models**

| **Predictor** | **Df** | **GVIF** | **GVIF^(1/(2·Df))** |
| --- | --- | --- | --- |
| Sex | 1 | 1.170 | 1.081 |
| Age | 1 | 1.202 | 1.097 |
| Disease duration | 1 | 1.050 | 1.025 |
| BMI | 1 | 1.200 | 1.095 |
| ALSFRS-R total score | 1 | 1.261 | 1.123 |
| FVC% predicted | 1 | 1.266 | 1.125 |
| Smoking status | 1 | 1.093 | 1.046 |
| Hypertension | 1 | 1.089 | 1.044 |
| Diabetes mellitus | 1 | 1.162 | 1.078 |

Note: GVIF diagnostics were calculated using the predictor matrix of the fully adjusted multivariable regression models. The model included sex, age, disease duration, BMI, ALSFRS-R total score, FVC% predicted, smoking status, hypertension, and diabetes mellitus. GVIF values were standardized as GVIF^(1/(2·Df)). Because all predictors in this model had one degree of freedom, GVIF^(1/(2·Df)) corresponds to the square root of GVIF. Because the same predictor set was used in the fully adjusted regression models across inflammatory and ultrasound outcomes, a single GVIF table was generated. Estimated progression rate was not included because it was derived from ALSFRS-R total score and disease duration. Other pulmonary function indices were not included because FVC% predicted was used as the pulmonary adjustment variable in the fully adjusted models. Outcome variables, including inflammatory markers and neuromuscular ultrasound measurements, were not included in the GVIF diagnostics because they served as dependent variables in the regression models. ALSFRS-R, Revised Amyotrophic Lateral Sclerosis Functional Rating Scale; BMI, body mass index; FVC, forced vital capacity; GVIF, generalized variance inflation factor.
