# Supplementary material for: Sex-associated differences in routine inflammatory markers and neuromuscular ultrasound measurements in amyotrophic lateral sclerosis: a retrospective cross-sectional study
Source: Ann Med. 2026 Jul 22;58(1):2703317. doi: 10.1080/07853890.2026.2703317 (PMC13393054; doi:10.1080/07853890.2026.2703317)
Supplement: Supplementary_Table_S2.docx [file IANN_A_2703317_SM1939.docx]

# Supplementary Table S2. Restricted cubic spline-adjusted sensitivity analysis for the sex effect

| Outcome | N | Linear β (95% CI) | Linear P value | RCS β (95% CI) | RCS P value | RCS interpretation |
| --- | --- | --- | --- | --- | --- | --- |
| NEU | 135 | -0.655 [-1.096, -0.214] | 0.004 | -0.698 [-1.150, -0.246] | 0.003 | Direction and significance retained |
| MONO | 135 | -0.092 [-0.133, -0.050] | <0.001 | -0.085 [-0.127, -0.043] | <0.001 | Direction and significance retained |
| USBT | 135 | -0.233 [-0.456, -0.010] | 0.041 | -0.201 [-0.427, 0.024] | 0.080 | Direction retained but significance attenuated |
| USMNCA | 135 | -0.016 [-0.026, -0.005] | 0.005 | -0.016 [-0.027, -0.005] | 0.005 | Direction and significance retained |
| USTLFRMCA | 135 | -1.202 [-2.196, -0.208] | 0.018 | -1.132 [-2.159, -0.105] | 0.031 | RCS significance retained; exploratory secondary finding |

This sensitivity analysis evaluated whether the sex-associated differences observed in the fully adjusted linear models remained stable after flexible modeling of continuous covariates using restricted cubic splines. The tested outcomes were NEU, MONO, USBT, USMNCA, and USTLFRMCA. In the primary fully adjusted linear model, age, disease duration, BMI, ALSFRS-R, and FVC% were modeled as linear terms. In the RCS-adjusted model, these continuous covariates were modeled using restricted cubic splines with three knots. Both models included sex, smoking status, hypertension, and diabetes. Male patients were used as the reference group. Beta values represent the adjusted difference in female patients compared with male patients. Although USTLFRMCA retained statistical significance in the RCS-adjusted model, it was interpreted as an exploratory secondary finding because the associations were not statistically significant in Models 1 and 2.
